# The differences in healthcare utilization for dental caries based on the implementation of water fluoridation in South Korea

**DOI:** 10.1186/s12903-016-0311-z

**Published:** 2016-11-08

**Authors:** Myung-Soo Cho, Kyu-Tae Han, Sohee Park, Ki Tae Moon, Eun-Cheol Park

**Affiliations:** 1 Department of Health Policy and Management, Graduate School of Public Health, Yonsei University, Seoul, Republic of Korea; 2 Division of Management & Planning, Korea Health Promotion Foundation, Seoul, Republic of Korea; 3 Department of Public Health, Graduate School, Yonsei University, Seoul, Republic of Korea; 4 Institute of Health Services Research, Yonsei University College of Medicine, Seoul, Republic of Korea; 5 Department of Biostatistics, Graduate School of Public Health, Yonsei University, Seoul, Republic of Korea; 6 National Evidence-Based Healthcare Collaborating Agency, Seoul, Republic of Korea; 7 Department of Preventive Medicine, Yonsei University College of Medicine, Seoul, Republic of Korea

**Keywords:** Water fluoridation, Dental caries, Healthcare expenditures, Public program

## Abstract

**Background:**

There were some debates about the water fluoridation program in South Korea, even if the program had generally substantial effectiveness. Because the out-of-pocket expenditures for dental care were higher in South Korea than in other countries, an efficient solution was needed. Therefore, we examined the relationship between the implementation of water fluoridation and the utilization of dental care.

**Methods:**

We used the National Health Insurance Service National Sample Cohort. In this study, data finally included 472,250 patients who were newly diagnosed with dental caries during 2003–2013. We performed survival analysis using cox proportional hazard model, negative binomial-regression, and regression analyses using generalized estimating equation models.

**Results:**

There were 48.49 % outpatient dental care visit during study period. Individuals with water fluoridation had a lower risk of dental care visits (HR = 0.949, 95 % CI = 0.928–0.971). Among the individuals who experienced a dental care visit, those with water fluoridation program had a lower number of dental care visits (β = −0.029), and the period of water fluoridation had an inverse association with the dental care expenditures.

**Conclusion:**

The implementation of water fluoridation programs and these periods are associated with reducing the utilization of dental health care. Considering these positive impacts, healthcare professionals must consider preventive strategies for activating water fluoridation programs, such as changes in public perception and relations, for the effective management of dental care in South Korea.

## Background

Dental caries are the most common diseases related to oral health worldwide [1]. The insufficient management of oral health to prevent dental caries could cause several symptoms and large cost burdens associated with the treatment of dental caries. Naturally, many strategies for preventing dental caries were developed, such as the improvement of oral health behaviors, changes in dietary patterns, the brushing of teeth, the use of fluoride toothpaste, flu dental screenings, dental scaling, and water fluoridation programs [2–4]. Recently, the oral health, especially that related to dental caries, of individuals has been generally improved compared with that in the past [5].

Water fluoridation is a program that introduces fluoride at an optimal level into drinking water to improve pubic dental health. It was first introduced in Grand Rapids in 1945 and was considered effective in the prevention of dental caries [6]. A previous report by the World Health Organization (WHO) suggested that the program could positively affect oral health, and the WHO recommended introducing water fluoridation in countries where such programs would be technically and culturally feasible [7]. Hence, many countries began to introduce water fluoridation. In the previous study which was conducted based on systematic review in UK, the water fluoridation had positive impact for reducing dental caries about 14 % [8]. This program in New Zealand and Australia also made about positive outcomes in oral health related to dental caries [9, 10]. The result in Japan also showed that positive effect too [11].

Since 1981, South Korea has implemented and gradually expanded a water fluoridation program. In 2002, the 32 regions among the ~250 regions in South Korea had implemented water fluoridation programs [12]. Since the late 1990s, some concerns about adverse effects of water fluoridation have been generally raised through media sources in South Korea. Each local government, with the support of voters, faces choices in its decision making for the implementation of water fluoridation. The expansion of water fluoridation thus faces difficulties, even if the program is effective in aspects of both quality of life and costs. Finally, only 17 regions implemented water fluoridation in 2013. Previous studies suggest, however, that there have been no significant adverse effects of introducing water fluoridation since the beginning of the 21st century [7, 8, 13].

Because of the effectiveness of water fluoridation and the continuous progress against dental caries in South Korea (age-adjusted prevalence rates of dental caries: 39.1 % in 2007, 31.4 % in 2014), we assumed that water fluoridation programs could significantly affect the reduction of dental caries in South Korea [14]. Although some previous studies in South Korea examined the effectiveness of water fluoridation, there were no studies took the perspective of the whole nation in South Korea. Also, the cost burden, including out-of-pocket expenses, for dental care is one of the major causes of medical expenditures (2011: Organization for Economic Co-operation and Development [OECD] average = 55 %, South Korea = 84 %) [15]. Therefore, we examined the relationship between the implementation of water fluoridation and the utilization of dental care using national sample cohort data. Based on our findings, we expect that decision makers could make appropriate strategies for the management of oral health in South Korea.

## Methods

### Study population

The data used in this study was the National Health Insurance Service National Sample Cohort 2002–2013. It include a random sample of 1,025,340 individuals as about 2.2 % of the overall South Korean in 2002. To represent total medical expenditure per year of South Korean within each of 1,476 strata (defined by age, sex, types of insurance coverage, and income level), this data was made through using probability sampling methods, and the model used proportional allocation. Thus, total medical claims of 1,025,340 individuals during 2002–2013 was included in this data. To examine the association between the implementation of water fluoridation program and the utilization of outpatient care for dental caries, represented by factors such as the frequency of outpatient visits and the medical expenditures by individuals, we only included patients who lived in non-metropolitan areas, because water fluoridation programs were rarely implemented in metropolitan areas (one metropolitan area in 2013). Then, we included only the patients who were newly diagnosed with dental caries (International Classification of Diseases [ICD]-10: K02) through outpatient care after 2003 (589,346 patients). We excluded those who were first diagnosed with dental caries before 2003 to ensure that only newly diagnosed patients were included. These patients were annually followed up. Finally, the data included 472,250 patients in 164 regions during 2003–2013 (average 9.12 years follow up). In addition, we added information about which regions had implemented water fluoridation from the Ministry of Health & Welfare. Regional variables were obtained from the ‘e-provincial indicators’ which was collected by Statistics Korea. It contained the regional characteristics of about 250 Si-Gun-Gu in South Korea. This data was used in this study for reflecting regional characteristics for each region where the patients with dental caries lived.

### Variables

The outcome variables used in this study were based on information about healthcare utilization for dental caries within the study population. The first outcome variable used in this study was whether each patient visited to dental clinic due to dental caries through outpatient care during study period. Next, other outcome variables were used in this study were the frequencies of dental care visits and the cost of outpatient care as indicators of medical expenditures for only the patients who had experienced outpatient care for dental caries in each year.

The major variables of interest were whether or not each community implemented water fluoridation during the study period and how long the period of the program was. Based on 2013, the water fluoridation program was implemented in 13 Si-Gun-Gu among 164 non-metropolitan areas. In this study, the patients who lived in those regions were considered to live in regions where water fluoridation had been implemented. Additionally, to consider the effect of the time since the initial implementation the program, the time since the implementation of water fluoridation was also included in this study. We hypothesized that the implementation of water fluoridation and the time since the implementation in each region would affect the outpatient visits and medical expenditures by the patients.

The patients and regional variables were controlled in analyses for association between water fluoridation and the outcome variables. The patient variables were as follows: age, sex, income, type of insurance coverage, study year, dental care expenditures in the previous year, dental care visits in the previous year, dentofacial anomalies, and disorders of tooth development and eruption. The patients’ age was categorized based on 10 years. The income was divided into deciles based on mean household income as follows: ≤10, 11–20, 21–30, 31–40, 41–50, 51–60, 61–70, 71–80, 81–90, and ≥91 %. The types of insurance coverage were categorized based on the NHI criteria as: medical aid, National Health Insurance (NHI) employee insurance, or NHI self-employed insurance. First, medical aid was defined as individuals with income less than poverty level in South Korea or individuals with a physical or mental disability. They could receive the healthcare services as free or low copayment by government funds. And, the other individuals were defined as NHI beneficiaries. The NHI beneficiaries were categorized in employee or self-employed based on job status. If individuals included workers and employers in all workplaces, they were included to NHI employee insurance, and paid a premium of about 7 % in their monthly income as withholding tax.The NHI self-employed insurance defined as individuals who did not fall into the NHI employee insurance group. They paid NHI premium based on their income, property, and living standard. Thus, this category could reflect the socio-economic status of each individual. We also included the number of pre-dental care visits, the pre-dental care cost, dentofacial anomalies (ICD-10: K07), and disorders of tooth development and eruption (ICD-10: K00) to adjust the severity of disease which could affect to the risk in dental caries in each patient. The number of pre-dental care visits was defined as the number of outpatient visits related to dental care during the previous year after the baseline year. The pre-dental care costs were calculated as the sum of the healthcare expenditures for dental care during the previous year after the baseline year. Dentofacial anomalies and disorders of tooth development and eruption were defined based on whether the patient was diagnosed with those symptoms as comorbidities in a specific year. The regional variables were the number of dentists per 1,000 people and the financial independence rate of the local government. The financial independence rate of the local government is an index of the finance utilization capacity of a local government with independent discretionary power. It was calculated as follows: (local taxes + non tax revenue)/budgets of local government × 100.

### Statistical analysis

First, we showed the frequencies of categorical variable and averages of continuous variable to examine the distribution of general characteristics at the baseline. We also performed χ^2^ tests and Cochran–Mantel–Haenszel tests to investigate the association between each categorical variables and visiting dental care by the implementation of water fluoridation as unit of person-years during study period. Next, we showed the averages and standard deviation of each continuous variable at the baseline and also performed a Mann-Whitney test, a Kruskal-Wallis test, and an analysis of covariance for continuous variable by the water fluoridation program during the study period. We also showed the Kaplan-Meier survival curve to compare the risk for visiting dental care by the water fluoridation. Finally, we performed a survival analysis using cox proportional hazard model to investigate whether patient visited to dental clinic due to dental caries during study period. Then, for the patients who had experienced outpatient care for dental caries during the study period, to examine the associations with the number of dental care visits and the dental care expenditures, we performed a multiple negative binomial regression, and regression analysis using a Generalized Estimating Equation (GEE) model with link logit regarding the overdispersion of outcome variables [16]. Additionally, we performed subgroup analyses according to age group or experience of pre-dental care within each region to examine the difference of association related to water fluoridation. The statistical analyses in used this study were analyzed using SAS statistical software version 9.4.

## Results

The data included 472,250 patients at baseline. Appendix 1 shows the general characteristics of the study population at baseline. The patients with water fluoridation were 10.40 % of the total patients at baseline. The average follow-up time was 9.12 years during the study period.

Table 1 shows the associations among the patient and regional characteristics of baseline, the outpatient dental care visits for dental caries, and the presence of water fluoridation during the study period. The average percentage of patients that experienced an outpatient dental visit was 46.98 % in regions with water fluoridation and 48.66 % in regions without water fluoridation, respectively (*p*-value < .0001). The distributions of the other independent variables based on water fluoridation were similar among the groups. Females more frequently experienced outpatient dental care than males (*p*-value < .0001). In addition, younger or wealthier groups were generally more likely to make dental visits because of dental caries than other groups (*p*-value < .0001). Beneficiaries of NHI employee insurance more visited to dental care than individuals with other types of insurance (*p*-value < .0001).

**Table 1:** The association between baseline characteristics and dental care visit during study period by water fluoridation program

Variables	Water flouridation										*P*-value
	Yes					No					
	Visit		Non-visit		*P*-value					*P*-value	
	N/Mean	%/SD	N/Mean	%/SD		N/Mean	%/SD	N/Mean	%/SD		
*Regional variables*											
Period from introduction of water fluoridation	6.40	±4.67	6.64	±4.74	<.0001	–	–	–	–	–	–
Number of dentists per 1,000 residents	0.29	±0.15	0.29	±0.15	<.0001	0.28	±0.12	0.27	±0.12	<.0001	0.0021
Financial independence rate of local government	46.48	±23.11	47.56	±23.36	<.0001	51.77	±24.63	50.36	±24.45	<.0001	<.0001
*Individual variables*											
Sex											
Male	10,934	44.14	13,837	55.86	<.0001	98,496	45.88	116,178	54.12	<.0001	<.0001
Female	12,142	49.86	12,209	50.14		107,402	51.52	101,052	48.48		
Age (Years)											
-19	8,880	59.43	6,063	40.57	<.0001	78,779	61.62	49,071	38.38	<.0001	<.0001
20–29	3,546	51.14	3,388	48.86		31,139	51.96	28,790	48.04		
30–39	3,663	43.75	4,710	56.25		32,436	45.26	39,233	54.74		
40–49	3,290	43.90	4,205	56.10		29,275	44.93	35,884	55.07		
50–59	1,872	43.30	2,451	56.70		16,961	44.98	20,743	55.02		
60–69	1,366	34.45	2,599	65.55		12,956	37.25	21,824	62.75		
< 70	459	14.86	2,630	85.14		4,352	16.71	21,685	83.29		
Income (percentile)											
-10 % (low)	1,911	35.95	3,405	64.05	<.0001	15,930	35.78	28,588	64.22	<.0001	<.0001
11–20 %	1,404	43.58	1,818	56.42		12,124	44.91	14,874	55.09		
21–30 %	1,705	44.68	2,111	55.32		14,157	46.55	16,253	53.45		
31–40 %	2,014	45.56	2,407	54.44		16,787	46.70	19,163	53.30		
41–50 %	2,289	46.19	2,667	53.81		19,872	48.74	20,899	51.26		
51–60 %	2,518	47.58	2,774	52.42		21,769	49.95	21,811	50.05		
61–70 %	2,818	49.68	2,854	50.32		24,962	50.83	24,148	49.17		
71–80 %	2,988	50.50	2,929	49.50		27,172	52.05	25,032	47.95		
81–90 %	2,840	50.87	2,743	49.13		28,038	52.66	25,206	47.34		
+ 91 % (high)	2,589	52.55	2,338	47.45		25,087	54.13	21,256	45.87		
Types of insurance coverage											
Medical Aid	449	22.90	1,512	77.10	<.0001	3,868	23.24	12,778	76.76	<.0001	<.0001
NHI, self-employed insured	10,619	46.96	11,995	53.04		92,100	48.14	99,217	51.86		
NHI, employee insured	12,008	48.92	12,539	51.08		109,930	51.09	105,235	48.91		
Year of baseline											
2003	20,564	47.40	22,819	52.60	<.0001	180,731	49.01	187,996	50.99	<.0001	<.0001
2004	661	55.22	536	44.78		5,917	58.95	4,120	41.05		
2005	412	61.40	259	38.60		3,783	65.57	1,986	34.43		
2006	346	58.25	248	41.75		3,595	58.85	2,514	41.15		
2007	271	51.82	252	48.18		3,056	57.40	2,268	42.60		
2008	272	47.97	295	52.03		2,897	57.84	2,112	42.16		
2009	221	42.91	294	57.09		2,276	49.04	2,365	50.96		
2010	170	37.53	283	62.47		1,808	41.23	2,577	58.77		
2011	96	24.55	295	75.45		1,093	26.17	3,083	73.83		
2012	44	10.21	387	89.79		563	12.55	3,924	87.45		
2013	19	4.79	378	95.21		179	4.01	4,285	95.99		
Dentofacial anomalies											
Yes	13	100.00	0	0.00	0.0001	70	100.00	0	0.00	<.0001	<.0001
No	23,063	46.96	26,046	53.04		205,828	48.65	217,230	51.35		
Disorders of toothe development and eruption											
Yes	89	100.00	0	0.00	<.0001	792	100.00	0	0.00	<.0001	<.0001
No	22,987	46.88	26,046	53.12		205,106	48.56	217,230	51.44		
Total	23,07	46.98	26,046	53.02		205,898	48.66	217,230	51.34		<.0001

†The results of a Cochran–Mantel–Haenszel test to investigate the differences of distribution for categorical variables based on water fluoridation

Appendix 2 shows the Kaplan-Meier survival curve for time to first diagnosis of dental caries by the implementation water fluoridation. Individuals in regions with water fluoridation program was less likely to visit dental care than individuals in regions without program (*p*-value for log-rank test < .0001).

Table 2 shows the distribution based on water fluoridation of the average values and the standard deviation for dental care visits or cost per year among only the patients who made dental visits because of dental caries. The average number of dental care visits and the costs were lower among patients with water fluoridation than among those without the program (*p*-value < .0001).

**Table 2:** The averages and standard deviations of dental care visits and costs for patients who visited dental care through outpatient care†

Variables	Water flouridation						*P*-value	Water flouridation						*P*-value†
	Yes			No				Yes			No			
	Number of dental care visits		*P*-value	Number of dental care visits		*P*-value		Dental care costs (KRW)		*P*-value	Dental care costs (KRW)		*P*-value	
	Mean	SD		Mean	SD			Mean	SD		Mean	SD		
*Individual variables*														
Sex														
Male	0.28	±0.75	<.0001	0.30	±0.79	<.0001	0.9079	8485.36	±26,233.38	0.0167	8758.21	±27,035.59	<.0001	0.6855
Female	0.29	±0.77		0.30	±0.81			8567.78	±26,234.69		8790.68	±26,725.42		
Age (Years)														
-19	0.38	±0.90	<.0001	0.42	±0.95	<.0001	<.0001	12358.18	±32,803.66	<.0001	12790.26	±33,474.42	<.0001	0.2527
20–29	0.26	±0.72		0.27	±0.76			7478.72	±24,358.22		7636.75	±24,858.38		
30–39	0.22	±0.65		0.23	±0.67			6137.69	±20,783.50		6353.85	±21,809.12		
40–49	0.22	±0.63		0.24	±0.68			6118.31	±20,360.00		6575.26	±22,017.51		
50–59	0.25	±0.69		0.24	±0.68			7203.43	±22,910.40		7305.94	±23,947.33		
60–69	0.26	±0.77		0.27	±0.75			7729.45	±25,167.92		7685.72	±24,254.82		
< 70	0.26	±0.76		0.27	±0.76			7378.33	±23,917.36		7392.36	±23,288.21		
Income (percentile)														
-10 % (low)	0.25	±0.74	0.0003	0.27	±0.81	<.0001	0.2805	7745.45	±25,837.11	0.0003	7969.37	±26,342.94	<.0001	0.0164
11–20 %	0.28	±0.78		0.29	±0.81			8170.88	±26,355.37		8539.98	±26,535.13		
21–30 %	0.28	±0.79		0.29	±0.80			8300.89	±26,029.00		8530.71	±26,384.56		
31–40 %	0.26	±0.70		0.29	±0.80			7579.31	±24,080.07		8585.70	±27,179.61		
41–50 %	0.28	±0.76		0.30	±0.80			8685.57	±27,015.42		8710.41	±26,762.40		
51–60 %	0.29	±0.77		0.30	±0.80			8869.81	±27,295.76		8910.14	±27,358.22		
61–70 %	0.30	±0.78		0.31	±0.81			8887.83	±26,420.46		9195.21	±27,617.53		
71–80 %	0.29	±0.77		0.32	±0.81			8868.81	±26,543.89		9330.15	±27,794.34		
81–90 %	0.30	±0.79		0.32	±0.80			9128.50	±27,290.42		9128.64	±27,025.66		
+ 91 % (high)	0.28	±0.72		0.30	±0.76			8348.43	±24,746.39		8392.44	±25,500.98		
Types of insurance coverage														
Medical Aid	0.17	±0.65	<.0001	0.19	±0.73	<.0001	0.9777	5463.02	±24,295.15	<.0001	5569.94	±23,732.00	<.0001	0.6780
NHI, self-employed insured	0.27	±0.75		0.29	±0.79			8349.77	±25,975.77		8657.48	±26,710.94		
NHI, employee insured	0.29	±0.77		0.31	±0.81			8768.09	±26,464.81		8971.88	±27,079.50		
Index year														
2003	0.25	±0.61	<.0001	0.25	±0.63	<.0001	<.0001	8034.83	±24,159.19	<.0001	8080.83	±24,641.98	<.0001	0.0003
2004	0.25	±0.63		0.25	±0.62			8382.93	±26,010.94		8009.35	±24,967.53		
2005	0.25	±0.61		0.25	±0.63			8297.59	±25,261.94		8270.24	±25,774.14		
2006	0.25	±0.63		0.25	±0.63			8283.21	±25,874.99		8310.02	±26,017.55		
2007	0.29	±0.78		0.30	±0.82			8302.10	±25,361.18		8447.74	±25,742.34		
2008	0.33	±0.90		0.34	±0.93			8527.55	±25,215.56		8613.98	±25,737.46		
2009	0.32	±0.87		0.34	±0.91			8484.86	±25,234.96		8797.15	±26,417.00		
2010	0.30	±0.83		0.32	±0.88			8363.59	±26,338.25		8692.93	±26,825.06		
2011	0.31	±0.84		0.34	±0.90			8821.34	±27,704.86		9519.33	±28,487.64		
2012	0.30	±0.83		0.33	±0.88			9062.00	±28,211.94		9642.10	±29,482.37		
2013	0.29	±0.79		0.33	±0.87			9443.44	±29,389.48		10376.20	±31,264.12		
Dentofacial anomalies														
Yes	1.75	±1.32	<.0001	1.91	±1.42	<.0001	0.0774	60991.70	±60,760.65	<.0001	68584.30	±65,057.42	<.0001	0.0088
No	0.28	±0.76		0.30	±0.80			8503.58	±26,182.17		8742.72	±26,802.56		
Disorders of toothe development and eruption														
Yes	1.89	±1.43	<.0001	1.95	±1.39	<.0001	0.1953	70958.60	±56,207.42	<.0001	71028.40	±54,669.15	<.0001	0.8636
No	0.28	±0.75		0.29	±0.79			8287.70	±25,760.74		8526.49	±26,412.60		
Total	0.28	±0.76		0.30	±0.80		<.0001	8528.51	±26,234.03		8775.16	±26,874.15		<.0001

† The results of analysis of covariance to investigate the differences of distribution for continuous variables based on water fluoridation

Table 3 shows the results of the survival analysis using cox proportional hazard model, negative binomial regression, and regression analysis for dental care expenditures, represented by the number of visits or the costs, among only the patients that experienced a dental visit. Patient with water fluoridation program had higher risk in the dental care visit during study period (Yes = HR: 0.949, 95 % CI: 0.928–0.971, *p*-value < .0001). Female and younger people was had more tend to dental care visits due to dental caries (*p*-value < .0001). By the economic status, people with higher income or employee insured NHI had high risk in dental care visits than other groups (*p*-value < .0001). In addition, people with dental comorbidities was also more visited in dental care (*p*-value < .0001).

**Table 3:** The results of survival analysis, negative binomial regression, and regression for dental care visits and expenditures including both patient and regional characteristics

Variables	Dental care visits^a^				Only patient with dental care visit					
					Number of dental care visits^b^			Dental care costs (KRW)^c^		
	HR	95 % CI		*P*-value	β	SE	*P*-value	β	SE	*P*-value
*Regional variables*										
Water fluoridation										
Yes	0.949	0.928	0.971	<.0001	−0.029	0.014	0.0431	0.008	0.021	0.7097
No	1.000	–	–	–	Ref	–	–	Ref	–	–
Period from implementation of water fluoridation	0.998	0.995	1.001	0.2302	−0.003	0.001	0.0177	−0.006	0.002	0.0024
Number of dentists per 1,000 residents	1.007	0.975	1.041	0.6700	−0.045	0.016	0.0068	−0.026	0.024	0.2812
Financial independence rate of local government	0.999	0.999	0.999	<.0001	−0.003	0.000	<.0001	−0.003	0.000	<.0001
*Individual variables*										
Sex										
Male	0.795	0.788	0.801	<.0001	−0.051	0.004	<.0001	−0.071	0.005	<.0001
Female	1.000	–	–	–	Ref	–	–	Ref	–	–
Age (Years)										
-19	4.835	4.695	4.979	<.0001	0.408	0.010	<.0001	0.662	0.013	<.0001
20–29	3.069	2.977	3.163	<.0001	0.064	0.011	<.0001	0.020	0.014	0.1451
30–39	2.466	2.392	2.542	<.0001	−0.073	0.011	<.0001	−0.134	0.014	<.0001
40–49	2.502	2.427	2.579	<.0001	−0.069	0.011	<.0001	−0.101	0.013	<.0001
50–59	2.531	2.452	2.613	<.0001	−0.022	0.011	0.0487	−0.030	0.014	0.0316
60–69	2.044	1.978	2.112	<.0001	0.033	0.012	0.0045	0.055	0.016	0.0004
< 70	1.000	–	–	–	Ref	–	–	Ref	–	–
Income (percentile)										
-10 % (low)	0.751	0.736	0.767	<.0001	0.003	0.009	0.7721	−0.085	0.013	<.0001
11–20 %	0.751	0.736	0.767	<.0001	−0.004	0.009	0.6797	−0.094	0.013	<.0001
21–30 %	0.762	0.747	0.777	<.0001	−0.004	0.009	0.6740	−0.099	0.013	<.0001
31–40 %	0.770	0.756	0.784	<.0001	−0.026	0.008	0.0018	−0.121	0.012	<.0001
41–50 %	0.805	0.790	0.819	<.0001	0.006	0.008	0.4173	−0.069	0.012	<.0001
51–60 %	0.835	0.821	0.850	<.0001	0.011	0.008	0.1536	−0.045	0.012	0.0001
61–70 %	0.880	0.866	0.895	<.0001	0.028	0.007	0.0002	−0.007	0.011	0.5510
71–80 %	0.916	0.901	0.931	<.0001	0.030	0.007	<.0001	0.025	0.011	0.0267
81–90 %	0.943	0.928	0.959	<.0001	0.023	0.007	0.0007	0.025	0.011	0.0190
+ 91 % (high)	1.000	–	–	–	Ref	–	–	Ref	–	–
Types of insurance coverage										
Medical Aid	0.410	0.396	0.425	<.0001	−0.573	0.018	<.0001	−0.956	0.017	<.0001
NHI, self-employed insured	0.872	0.865	0.879	<.0001	−0.033	0.004	<.0001	−0.097	0.006	<.0001
NHI, employee insured	1.000	–	–	–	Ref	–	–	Ref	–	–
Year of baseline/Index year										
2003	1.000	–	–	–	Ref	–	–	Ref	–	–
2004	1.052	1.026	1.079	<.0001	−0.015	0.003	<.0001	−0.126	0.013	<.0001
2005	1.126	1.092	1.162	<.0001	−0.018	0.003	<.0001	−0.102	0.013	<.0001
2006	1.124	1.089	1.160	<.0001	−0.017	0.003	<.0001	−0.108	0.013	<.0001
2007	1.143	1.104	1.183	<.0001	0.128	0.004	<.0001	−0.037	0.013	0.0037
2008	1.104	1.065	1.144	<.0001	0.209	0.004	<.0001	0.016	0.013	0.2238
2009	1.038	0.997	1.080	0.0710	0.192	0.004	<.0001	0.000	0.013	0.9752
2010	0.974	0.931	1.018	0.2418	0.176	0.004	<.0001	−0.073	0.013	<.0001
2011	0.736	0.695	0.779	<.0001	0.163	0.004	<.0001	0.079	0.013	<.0001
2012	0.483	0.445	0.523	<.0001	0.157	0.004	<.0001	0.045	0.013	0.0007
2013	0.304	0.265	0.350	<.0001	0.149	0.004	<.0001	0.091	0.014	<.0001
Pre-dental care costs (per 1,000 KRW)					0.001	0.000	<.0001	0.004	0.000	<.0001
Number of pre-dental care					0.183	0.004	<.0001	0.309	0.009	<.0001
Dentofacial anomalies										
Yes	21.614	17.417	26.821	<.0001	1.925	0.022	<.0001	8.832	0.037	<.0001
No	1.000	–	–	–	Ref	–	–	Ref	–	–
Disorders of toothe development and eruption										
Yes	16.465	15.392	17.614	<.0001	1.563	0.008	<.0001	8.348	0.012	<.0001
No	1.000	–	–	–	Ref	–	–	Ref	–	–

^a^The survival analysis using cox proportional hazard models was applied to investigate the association with risk of dental care visits during study period

^b^The negative binomial regression using GEE models was performed for patients who had experienced a dental care visit at least once per year to investigate the associations with dental care visits

^c^The regression using GEE models was performed for patients who had experienced a dental care visit at least once per year to investigate the associations with dental care cost

The results of the regression analysis for dental care expenditures showed that patients in regions that implemented a water fluoridation program had a lower number of dental visits or lower costs, although the results about dental care cost was not statistically significant (number of dental visits: β = −0.029, *p*-value = 0.0431; dental care costs: β = −0.008, *p*-value = 0.7097). In addition, patients in regions with more dentists made less dental visits and spent less on dental care. On the other hand, the financial independence rate in each region had an inverse association with the risk of dental visits (*p*-value < .05). For the patient characteristics, males had an inverse association with the number of dental visits and costs (*p*-value < .0001). On the other hand, beneficiaries of Medical Aid or self-employed NHI had a lower number of dental visits and lower costs than beneficiaries of employee NHI (*p*-value < .05). Expenditures related to pre-dental care or comorbidity had a positive association with the outcome variables (*p*-value < .001).

We performed subgroup analyses for the survival analysis using cox proportional hazard model, negative binomial regression, and regression analyses using GEE models to investigate the relationship between water fluoridation and the outcome variables based on experience of pre-dental care visits (visit or no visit), income (−50 % and +51 %), and age group (≤19 years, 20–39 years, 40–59 years, ≥60 years). The associations between the presence of a water fluoridation program and the number of dental visits was greater for patients who had not experienced a pre-dental care visit in the previous year than for patients who had experienced a pre-dental care visit in the previous year (with, water fluoridation = β: 0.043, *p*-value = 0.2901; without, water fluoridation = β: −0.031, *p*-value = 0.0424). By age group, the inverse associations between water fluoridation and outcome variables were greater in the elderly population than in the younger population. In the younger groups, the inverse associations with dental care expenditures were analyzed based on period from implementation of water fluoridation (Table 4).

**Table 4:** The results of subgroup analysis for survival analysis, negative binomial regression, and regression by pre-dental care and patient age

Sub group		Variables	Dental visits^a^				Only patient with dental care visit					
							Number of dental care visits^b^			Dental care costs (KRW)^c^		
			HR	95 % CI		*P*-value	β	SE	*P*-value	β	SE	*P*-value
Experience of pre-dental care in previous year	With	Water fluoridation					0.043	0.041	0.2901	0.064	0.047	0.1712
		Period from implementation of water fluoridation					−0.007	0.004	0.0689	−0.003	0.004	0.4521
	Without	Water fluoridation					−**0.031**	**0.015**	**0.0424**	−0.009	0.018	0.6180
		Period from implementation of water fluoridation					−0.002	0.001	0.0959	**−0.004**	**0.002**	**0.0115**
Income (percentile)	−50 %	Water fluoridation	**0.934**	**0.900**	**0.968**	**0.0002**	**−0.0532**	**0.0247**	**0.0315**	−0.0106	0.0288	0.7131
		Period from implementation of water fluoridation	1.001	0.996	1.006	0.6484	−0.0004	0.0022	0.8424	−0.0026	0.0025	0.2927
	51 %	Water fluoridation	**0.957**	**0.930**	**0.985**	**0.0032**	−0.0083	0.0178	0.6422	0.0173	0.0212	0.4162
		Period from implementation of water fluoridation	0.997	0.993	1.000	0.0792	**−0.0050**	**0.0016**	**0.0014**	**−0.0056**	**0.0018**	**0.0020**
	−19	Water fluoridation	**0.941**	**0.906**	**0.976**	**0.0013**	−0.0086	0.0232	0.7117	0.0048	0.0276	0.8617
		Period from implementation of water fluoridation	0.996	0.992	1.001	0.1132	**−0.0070**	**0.0021**	**0.0007**	**−0.0054**	**0.0025**	**0.0270**
Age group	20–39	Water fluoridation	**0.959**	**0.922**	**0.998**	**0.0408**	−0.0113	0.0263	0.6675	0.0274	0.0309	0.3755
		Period from implementation of water fluoridation	1.001	0.996	1.006	0.8145	−0.0034	0.0023	0.1495	**−0.0068**	**0.0027**	**0.0104**
	40–59	Water fluoridation	**0.952**	**0.908**	**0.999**	**0.0461**	−0.0568	0.0301	0.0595	−0.0211	0.0352	0.5493
		Period from implementation of water fluoridation	1.000	0.994	1.006	0.9847	−0.0001	0.0026	0.9676	−0.0025	0.0029	0.3957
	60+	Water fluoridation	**0.912**	**0.840**	**0.991**	**0.0297**	−0.0518	0.0465	0.2648	0.0010	0.0568	0.9864
		Period from implementation of water fluoridation	0.998	0.987	1.010	0.7551	0.0022	0.0039	0.5666	0.0008	0.0048	0.8707

The reference level of water fluoridation was that of regions without a program, and the period since the implementation of water fluoridation was a continuous variable

The bold face indicates statistically significant results

These were results of sub group analyses for survival analysis and negative binomial regression analyses

^a^The survival analysis using cox proportional hazard models was applied to investigate the association with the risk of dental care visits

^b^The negative binomial regression using GEE model was performed for patients who had experienced a dental care visit at least once per year to investigate the associations with dental care visits.

^c^The regression using GEE model was performed for patients who had experienced a dental care visit at least once per year to investigate the associations with dental care costs

## Discussion

To effectively manage oral health, and particularly patients with dental caries in South Korea, the South Korean government has introduced water fluoridation programs since 1981 [12]. Previous studies of the water fluoridation programs showed that the programs had a positive role in preventing dental caries and improving oral health [10, 17, 18]. Based on those results, the water fluoridation programs seemed to gradually expand on the whole in South Korea. However, in the late 20th century, some concerns related to negative effects of water fluoridation, such as fluoride toxicity, were raised and proliferated. After that, the activation of the programs faced difficulties such as a negative public awareness. However, the previous studies of the adverse effects of water fluoridation found no significant adverse effects related to fluoride [7, 8, 13]. There was a need to change the public perception and provide evidence supporting the activation of water fluoridation programs for effective public health. Therefore, we examined the relationship between the implementation of water fluoridation and dental care utilization by South Koreans using a national sample cohort during 2003–2013.

Our findings showed that the implementation of the water fluoridation program in each region had a preventive role, decreasing dental care utilizations such as dental visits and expenditures. In the regions with water fluoridation programs, the duration of the program had an inverse association with the number of dental visits per year. Those findings were similar to the findings of previous studies including results in the other countries [8–11]. Water fluoridation programs as a prevention strategy could positively affect the improvement of oral health, and individuals who lived in regions with water fluoridation had less need to access dental care because of their improved oral health. The out-of-pocket expenditures for dental care in South Korea were remarkably higher than those in other OECD countries; introducing such prevention strategies had a quite positive affect on the reduction of the disease burden in South Korea [15]. Therefore, the implementation of water fluoridation improved oral health in South Korea, and a national recommendation for the activation of this program would be worthwhile based on our findings.

In addition, our study suggests some interesting results related to other covariates. The results for the financial independence rate of the local government as an indirect indicator of regional wealth revealed some inequalities in oral health among regions based on wealth, even if some community oral health programs were already implemented [19]. Hence, governments need to consider public health programs, such as regular dental screening programs, which could reduce inequalities. Also, the patients with a lower economic level, represented by income or type of insurance coverage, had a lower risk of dental visits and expenditures. Such results might be caused by the accessibility of health care. The wealthier individuals made more dental visits in the early stages of their clinical symptoms, and they used regular screening or consultation as part of primary and secondary prevention. On the other hand, individuals with low economic status could not make dental visits in the early stages of their symptoms and could not engage in prevention behaviors because of the cost burden [20, 21]. Therefore, the makers of health policy such as Korea Health Promotion Foundation must consider some interventions to reduce economic barriers for those with low economic status to reduce health inequalities caused by economic issues. Finally, there were results showing that younger patients or patients with comorbidities utilized more dental care. Those results were similar to previous results about dental care. That is because younger patients are at a period of change from primary teeth to permanent teeth, whereas the elderly had fewer teeth because of deformation [22, 23]. Also, the patients with comorbid conditions such as dentofacial anomalies or disorders of tooth development and eruption made more dental visits than patients without those problems [24, 25]. Such patients need to activate preventive healthcare to reduce preventable expenditures.

The reduction of dental care utilization by the introduction of water fluoridation showed some differences in the subgroup analyses. The results showed that such associations were greater among patients that had not experienced dental care. It was suggested that the water fluoridation program had a major role in the reduction of the incidence of dental caries as a primary prevention rather than a secondary prevention. The implementation of such programs would be effective for individuals who managed their dental care well without clinical symptoms. Therefore, healthcare professionals need to recommend the importance of managing oral health even for individuals without any symptoms [26].

Our study has some strengths compared with previous studies. First, we used national health insurance national sampling cohort data to analyze the relationship between the implementation of water fluoridation programs and dental visits or expenditures. Therefore, the data used in our study are especially helpful for establishing evidence-based health policy for dental care. Second, to our best knowledge, this study is the first attempt to investigate the impact of water fluoridation programs on the whole nation of South Korea. So far, several previous studies of water fluoridation have been conducted, but most of those only investigated the impact of the programs at the community level [27–29]. Therefore, our findings would be effective evidence for establishing public health programs or policies. Third, we included the dental visits and expenditures as outcome variables. Therefore, our results could reflect overall dental care utilization by each patient. Finally, we adjusted the data for pre-dental care and other comorbid conditions to provide a more detailed study. Thus, we could reflect the severity of the dental caries experienced by the patients in our analyses.

Our study had also some limitations. Based on previous studies of dental caries or oral health, various factors including lifestyles, types of tooth paste, and frequencies of tooth brushing could affect the health outcome [1, 30]. The impact of water fluoridation program could be different by cultural factors which affect to heathy behaviors in each countries. Also, the outcome variables in previous studies were used with other clinical indicators including the Patient Hygiene Performance index [31]. However, the relevant details were not included in the data, as the data used in our study had the characteristics of health insurance claim data. Therefore, we were unable to consider other variables that could affect dental caries. Next, we could not consider the fluoride level in the water or the actual consumption volume for each individual because of limitations of the data [32, 33]. In addition, we excluded the regions that quit the program during the study period, because we could not clearly identify the period effect after the introduction of water fluoridation. Therefore, more detailed studies are needed. Third, in the characteristics of dental care, there were many non-payment items related to dental care in South Korea. However, because of the characteristics of the NHI claim data, we could not consider those items. Thus, it is possible that the measurement of the dental care expenditures could have been underestimated. Finally, regarding some studies of water fluoridation programs that suggested that the programs had an adverse effect, there was a need to evaluate adverse effects. However, we only analyzed the dental care utilization and did not include any adverse effects.

Despite those limitations, our findings suggest that the implementation of water fluoridation programs had a positive role in improving oral health, and especially dental caries. Regarding the negative public perception of water fluoridation programs and the high out-of-pocket expenditures for dental care in South Korea, those findings could be helpful in the management of dental care expenditures from the perspective of health policy and public health. Although further detailed studies using clinical factors will be needed in the near future, makers of health policy and professionals in the area of dental care must consider efficient strategies for activating water fluoridation programs.

## Conclusions

The implementation of water fluoridation programs is inversely associated with the risk of dental visits and expenditures related to dental caries. Considering that association, makers of health policy and decision makers such as Korea Health Promotion Foundation have to consider preventive strategies for activating water fluoridation programs, such as changes in public perception and relations, in order to effectively manage dental care in South Korea.

## Figures and Tables

**Fig. 1:**
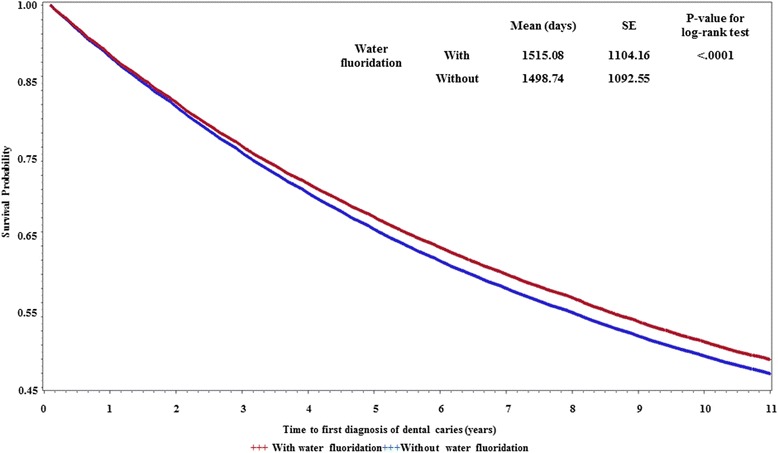
The Kaplan-Meier Survival curve for time to first diagnosis of dental caries by the implementation water fluoridation

## Appendix 1

**Table 5:** Characteristics of study population at baseline

Variables	N/Mean	%/SD
*Regional variables*		
Water fluoridation		
Yes (*n* = 13)	49,122	10.40
No (*n* = 151)	423,128	89.60
Period from implementation of water fluoridation	0.68	2.51
Number of dentists per 1,000 residents	0.28	0.12
Financial independence rate of local government	50.63	24.45
*Individual variables*		
Sex		
Male	239,445	50.70
Female	232,805	49.30
Age (Years)		
-19	142,793	30.24
20–29	66,863	14.16
30–39	80,042	16.95
40–49	72,654	15.38
50–59	42,027	8.90
60–69	38,745	8.20
> 70	29,126	6.17
Income (percentile)		
-10 % (low)	49,834	10.55
11–20 %	30,220	6.40
21–30 %	34,226	7.25
31–40 %	40,371	8.55
41–50 %	45,727	9.68
51–60 %	48,872	10.35
61–70 %	54,782	11.60
71–80 %	58,121	12.31
81–90 %	58,827	12.46
+ 91 % (high)	51,270	10.86
Types of insurance coverage		
Medical Aid	18,607	3.94
NHI, self-employed insured	213,931	45.30
NHI, employee insured	239,712	50.76
Year of baseline		
2003	412,110	87.27
2004	11,234	2.38
2005	6,440	1.36
2006	6,703	1.42
2007	5,847	1.24
2008	5,576	1.18
2009	5,156	1.09
2010	4,838	1.02
2011	4,567	0.97
2012	4,918	1.04
2013	4,861	1.03
Dentofacial anomalies		
Yes	83	0.02
No	472,167	99.98
Disorders of toothe development and eruption		
Yes	881	0.19
No	471,369	99.81
Follow up time (year)	9.12	2.80
Total	472,250	100.00

## Abbreviations

CI: Confidence interval
GEE: Generalized estimating equation
HR: Hazard ratio
ICD: International classification of diseases
KNHIS: Korean National Health Insurance Service
NHI: National Health Insurance
OECD: Organization for Economic Co-operation and Development
SE: Standard error
WHO: World Health Organization
